# Instrumental Activities of Daily Living by Subjective and Objective Measures: The Impact of Depression and Personality

**DOI:** 10.3389/fnagi.2022.829544

**Published:** 2022-07-22

**Authors:** Katya Numbers, Sujin Jang, Henry Brodaty, Perminder S. Sachdev, Brian Draper, Simone Reppermund

**Affiliations:** ^1^Centre for Healthy Brain Ageing, Discipline of Psychiatry and Mental Health, Faculty of Medicine and Health, University of New South Wales, Sydney, NSW, Australia; ^2^Department of Developmental Disability Neuropsychiatry, Faculty of Medicine and Health, School of Psychiatry, University of New South Wales, Sydney, NSW, Australia

**Keywords:** IADL, functional ability, depression, openness, neuroticism, conscientiousness

## Abstract

**Objective:**

Previous research shows that depression and personality are independently associated with self- and informant-reports of the ability to perform instrumental activities of daily living (IADLs). However, less is known about the association between depression and personality and performance-based measures of IADLs. We aimed to determine how depression and personality predict self-and informant-reports of IADL compared to performance-based measures of IADLs in a sample of older adults with normal cognition (NC) and Mild Cognitive Impairment (MCI).

**Methods:**

Participants consisted of 385 older adults with NC (*n* = 235), or a diagnosis of MCI (*n* = 150), aged between 76 and 99-years from the Sydney Memory and Ageing Study. Participants underwent comprehensive neuropsychological and clinical assessments to determine global cognition and clinical diagnoses. Personality traits were measured by the NEO Five-Factor Inventory (NEO-FFI) and depression by the Geriatric Depression Scale (GDS). Subjective IADLs were self- and informant-reported Bayer Activities of Daily Living (B-ADL) scales and objective IADL was the Sydney Test of Activities of Daily Living in Memory Disorders (STAM). Linear regressions examined the relationship between depression and personality and the three types of IADL measures, controlling for all covariates and global cognition.

**Results:**

Participant-reported IADL, although associated with global cognition, was more strongly associated with GDS and NEO-FFI scores (conscientiousness and neuroticism). Informant-reported IADL was strongly associated with both global cognition and participants’ GDS scores. STAM scores were not associated with participants’ GDS or NEO-FFI scores; instead, they were predicted by demographics and global cognition.

**Conclusion:**

These results suggest that performance-based measures of IADL may provide more objective and reliable insight into an individual’s underlying functional ability and are less impacted by the participants’ mood and personality compared to subjectively reported IADL. We argue that performance-based IADL measures are preferable when trying to accurately assess everyday functional ability and its relationship to cognitive status. Where performance-based measures are not available (e.g., in some clinical settings), informant ratings should be sought as they are less influenced by the participant’s personality and mood compared to self-reports.

## Introduction

The cornerstone of functional independence among older adults is an intact ability to perform necessary activities of daily living (ADL). The loss of independence in these activities is a key factor affecting the quality of life in individuals with dementia and their caregivers (Kempen et al., 1997). Impairment in ADL is a key feature in the diagnosis of dementia, with loss of ability to perform basic activities of daily living (BADL), or well-rehearsed everyday tasks such as ambulating, toileting, bathing, grooming, and feeding (Mlinac and Feng, 2016), distinguishing mild dementia from moderate or severe dementia according to the 5th edition of the Diagnostic and Statistical Manual of Mental Disorders (DSM-5) (Mitchell and Miller, 2008). Instrumental activities of daily living (IADL), such as driving, shopping, and managing finances or medications (Gold, 2012), recruit multiple cognitive domains and require planning and cognitive flexibility to complete (Mitchell and Miller, 2008). IADL is mostly preserved in Mild Cognitive Impairment (MCI) (Teng et al., 2010) but becomes impaired to varying degrees in mild dementia. The reliable and accurate assessment of IADL in an individual with cognitive deficits is therefore critical for determining a dementia diagnosis.

Currently, the most common method for assessing IADL is self- or informant- (i.e., close friend/family member) reported questionnaires (Tabert et al., 2002), which are quick and easy to collect and require relatively few resources or training to administer. Evidence suggests that, when given the opportunity for multiple observations, subjectively reported IADL gives a reasonably accurate representation of real-world performance (Schmitter-Edgecombe et al., 2011). However, a downside to self-reported measures of IADL is that they are based on people’s perceptions of their own functioning, which may result in overestimation or underestimation of actual ability (Coman and Richardson, 2006), especially as poorer awareness of IADL difficulties is associated with cognitive impairment (Albert et al., 1999; Steward et al., 2019). Further, self-reported IADL is known to be affected by an individual’s mood and personality traits. Specifically, individuals experiencing depressive symptoms (Tas̨ et al., 2007; Karakurt and Unsal, 2013; de Paula et al., 2015; Storeng et al., 2018) tend to overreport impairments in IADL, as do those who score higher on neuroticism (Krueger et al., 2006) and lower on conscientiousness (Suchy et al., 2010) according to a five-factor personality model [e.g., NEO-Five Factor Inventory (Costa, 1992)]. Informant-reported IADL is not subject to many of these limitations and therefore is more frequently used to assess participants’ functional capacity in research and clinical settings. However, informant-reported IADL can also be impacted by the participant’s depressive symptoms or personality traits, as some studies have shown (Votruba et al., 2015). Other factors such as informants’ own depressive symptoms and personality (Argüelles et al., 2001; Pfeifer et al., 2013), perceived burden of caring for the participant (Zanetti et al., 1999), social desirability and halo effects (Pereira et al., 2010), and limited insight into the daily routines of the participant (Martyr et al., 2014), can influence informant-reports of IADL as well.

To provide a more objective alternative to subjective self- and informant-reported IADL, performance-based measurements have been developed (Zanetti et al., 1999). Performance-based measures require participants to perform various IADL activities, such as measuring out medications or counting money for shopping, under direct observation from the assessor (Sikkes and Rotrou, 2014). Performance-based measures are less subject to bias, lack of insight, and the informant’s knowledge of and feelings toward the individual (Zanetti et al., 1999; Griffith et al., 2003; Goldberg et al., 2010). Therefore, performance-based IADLs are more sensitive to subtle decrements in IADL function (Goldberg et al., 2010; Pereira et al., 2010). However, performance-based measures are more time-consuming and expensive (Moore et al., 2007) and require specialized materials and training to administer (Reppermund et al., 2017). Moreover, it is possible that an individual’s level of depression and personality factors may influence their performance on objective measures of IADL as well.

While some studies have examined the association between personality and self-report vs. performance-based IADL (Suchy et al., 2010), to our knowledge, no study has investigated the relationship between depressive symptoms and personality and scores on a self-report, informant-report, and performance-based measure of IADL concurrently. It is important to understand this relationship clearly to determine whether interventions that target depression, a modifiable condition, may help to preserve functional capacity in older adults without a dementia diagnosis (Albert et al., 1999). Moreover, it is necessary to clarify how this relationship differs for performance-based IADL measures, as these have been shown to be more sensitive in detecting subtle IADL impairments and predicting cognitive decline (Triebel et al., 2009; Puente et al., 2014; Sikkes and Rotrou, 2014). The aim of the current study was to assess whether participants’ cognitive status, current depressive symptoms, and personality traits were differentially associated with IADL scores captured by subjective (self-report and informant-report) and objective (performance-based) measures, and whether this association captures variance above and beyond demographics and potential medical confounders.

## Materials and Methods

### Participants

The present study reports data from participants in the Sydney Memory and Ageing Study (MAS), a longitudinal study of community-dwelling older adults aged 70–90 years that began in 2005 (Sachdev et al., 2010). Of the 8,914 individuals invited to participate, 1,037 participants were included in the baseline sample. Inclusion criteria were the ability to speak and write English sufficiently well to complete a psychometric assessment and self−report questionnaires. MAS baseline exclusion criteria were major psychiatric diagnoses, acute psychotic symptoms, or a current diagnosis of multiple sclerosis, motor neuron disease, developmental disability, progressive malignancy, or dementia. More detailed methods of recruitment and baseline demographics have been previously described by Sachdev et al. (2010).

Every 2 years, MAS participants undertook a detailed assessment with a trained research assistant during which they completed a comprehensive neuropsychological test battery, medical history, medical exam, and a series of questionnaires. Clinical diagnoses were made at each by an expert consensus panel who considered all available neuropsychological, clinical, and imaging data. At a 6-year follow-up, MCI was diagnosed using international consensus criteria (Winblad et al., 2004), and dementia was diagnosed according to the Diagnostic and Statistical Manual of Mental Disorders (DSM-IV) (American Psychiatric Association, 1980), though participants meeting the later consensus criteria were excluded from the present analysis (see below).

For the present study, data from the 6-year follow-up are considered as this was the first time the performance-based assessment of IADL was administered. Of the 708 participants from the original MAS baseline sample included in the 6-year follow-up, there were 478 that completed the performance-based IADL measure. For the present study, further exclusion criteria were a consensus diagnosis of dementia at 6-year follow-up (*n* = 38), as such a diagnosis precluded administration of both the self-report and the performance-based IADL measures, and a non-English speaking background (i.e., not speaking English at a basic conversational level by the age of 9; *n* = 55), as cultural and linguistic variables, can bias standardized neuropsychological test results (Kochan et al., 2010). Thus, the final sample for this study comprised 385 participants, all of whom had an informant. Informants were friends or family members nominated by the participant who answered questions relating to the participant’s memory, thinking, and daily functioning. Informants were required to have at least 1 h of contact with the participant per week, but reported, on average closer to 6 h (*M* = 5.89, *SD* = 8.12) of contact per week.

All participants and informants provided written informed consent to participate in this study, which was approved by the University of New South Wales Human Ethics Review Committee (HC:09382, 14327).

### Measures

#### Self-Reported Instrumental Activities of Daily Living Assessment

To directly compare participants- and informant-reported IADL, we created a modified version of the Bayer-IADL. Where the original Bayer-IADL is designed to be administered to informants, we changed the pronoun of each question from third person to first person (e.g., “Does the *participant* have difficulty managing *his/her* medications?” to “Do *you* have difficulties managing *your* medications?” In this way, a direct comparison of participants’ and informants’ subjective appraisal of the same everyday activities could be captured while avoiding discrepancies between item phrasing or functional domains across measures. The modified self-report Bayer-IADL also takes on average 5 min to complete and is comprised of the same 25 items as the original Bayer-IADL (Hindmarch et al., 1998), which are also summed and divided by the number of items rated by the participant, with higher scores indicating more severe deficits.

#### Informant-Reported Instrumental Activities of Daily Living Assessment

The Bayer-Activities of Daily Living Scale (Bayer-IADL) (Hindmarch et al., 1998) was originally developed as an informant-based instrument to assess functional ability in the early stages of MCI and dementia. The Bayer-IADL typically takes 5 min to complete and is comprised of 25 items subdivided into three major areas: general ability to perform self-care and manage everyday activities (2 items), ability to perform specific everyday activities (18 items), and cognitive functions important for managing everyday life (5 items). Each item is introduced with the statement “Does the participant have difficulty…” and scores range from 1 (never) to 10 (always), with a “not applicable” or “unknown” option for each item. Scores are summed and divided by the number of items scored by the informant (i.e., not scored as “unknown” or “not applicable”), with higher scores indicating more severe deficits.

#### Performance-Based Instrumental Activities of Daily Living Assessment

The Sydney Test of Activities of daily living in Memory disorders (STAM) (Reppermund et al., 2017) is a performance-based measure of the ability to carry out a range of IADL. The STAM consists of nine items assessing the following domains of function: communication (making a phone call), dressing (putting on a shirt), handling finances (paying a bill by check), managing everyday activities (preparing the check for mailing), orientation to time (reading the time and setting an alarm), medication management (dispensing weekly medications), shopping (choosing items to make a simple recipe), counting money (calculating cost and counting money), and memory (recalling completed STAM activities). Each item on the STAM is scored on a 4-point scale, such that participants receive 1 point for each component completed correctly, for a maximum of 36 points where higher scores represent better performance. Each task also has a time limit, whereby participants were penalized if they went over time according to bracketed upper- and lower- time cut-offs, with the STAM taking on average 15 min to complete. The complete scale, including instructions and item components for scoring, is provided as Supplementary Material.

#### Objective Cognitive Performance

Global cognition composite scores were based on participants’ scores on a comprehensive neuropsychological test battery comprised of 10 tests that measured the domains of attention, language, executive function, visuospatial ability, memory, and verbal memory. Global cognition composite scores are presented as standardized z-scores and were derived as follows: Raw neuropsychological test scores were first converted to z-scores using the means and standard deviations (SDs) of a reference group comprised of 723 MAS participants from baseline that were classified as cognitively healthy (i.e., native English speakers with an MMSE score of 24 or above, no evidence of dementia or current depression, no history of delusions or hallucinations, and no major neurological disease, or significant head injuries). Composite domain scores were formed by averaging the z-scores of the component tests. Global cognition scores at each wave were calculated by averaging the domain scores. Global Cognition scores were standardized against the mean and SD (0 and 1, respectively) of the baseline reference group. More details about how cognitive domain and global cognition scores were calculated, and which tests comprised each cognitive domain, can be found in Supplementary Material.

#### Depression and Personality

The short form of the Geriatric Depression Scale (GDS) (Sheikh et al., 1986) was administered to assess participants’ current depressive symptoms. The GDS is a self-reported measure that requires a yes/no response to 15 questions about current mood, with higher scores indicating more depressive symptoms. A score of 5 is the recommended cut-off for clinically relevant depression (Pocklington et al., 2016). We used the GDS version with item 9 as described in Brink (here item 12) (Brink, 1982). Personality traits were assessed using a modified 36-item version of the NEO Five-Factor Inventory NEO-FFI (Costa, 1992). The original NEO-FFI is a 60-item questionnaire that assesses the big five personality traits of Extraversion, Agreeableness, Openness, Neuroticism, and Conscientiousness. For the present study, only the latter three personality traits were considered. This decision was made to reduce participant burden at baseline and based on existing evidence suggesting Openness, Neuroticism, and Conscientiousness are most highly correlated with subjective cognitive complaints and incident dementia (Comijs et al., 2002; Duchek et al., 2007; Wilson et al., 2007). Twelve items relate to each of the three personality traits. Participants were asked to rate the degree to which they agree with each statement as it relates to their own beliefs or attributes on a 5-point scale, with higher scores indicating a higher prevalence of each personality trait.

#### Covariates

Covariates included basic demographics of age, sex, and education. Medical covariates of interest comprised self-reported arthritis and vision impairment, which we transformed into a composite variable given both impairments have been shown to significantly impact participants’ ability to complete performance-based measures of IADL (Reppermund et al., 2017). Additionally, we included participants’ self-reported number of medications (comprised of both prescription and over-the-counter medications), as a proxy for medical comorbidities, as well as the Framingham Risk Scores (D’Agostino et al., 2008) to capture cardiovascular disease (CVD) risk.

#### Statistical Analysis

Prior to analyses, all variables were screened for violations of the assumptions associated with univariate and multivariate tests. Both STAM and informant-reported Bayer-IADL scores were non-normally distributed; to avoid potentially inflating α, these scores were transformed (LOG_10_), to improve normality and linearity, and univariate outliers were Windsorized. As analyses for both the raw and the transformed STAM and informant-report Bayer-IADL variables produced equivalent results, the raw data are listed in the table for ease of interpretation, but the transformed variables were used in analyses. Correlational analyses evaluated the relationship between participant-reported Bayer-IADL, informant-reported Bayer-IADL, and performance-based STAM scores.

Three hierarchical linear regressions were run to determine the predictive ability of participants’ depression and personality traits on self-report, informant-report, and performance-based IADL scores, over and above the variance assumed by participant demographics, and medical covariates, and global cognitive function. For each of the three IADL outcome measures, a three-step hierarchical regression model was run in the same order. Step 1 always included participants’ demographics (age, sex, and education) and medical comorbidities (vision impairment *and* arthritis, CVD risk score, total number of medications); Step 2 additionally included participants’ Global Cognition composite score; and Step 3 additionally included participants’ depression (GDS) and personality (NEO-FFI; neuroticism, conscientiousness, openness) scores. We ran additional *post-hoc* analyses to determine whether diagnostic status (i.e., NC vs. MCI) impacted the pattern of results. To do this, we ran the same series of hierarchical regressions, using the same steps and covariates outlined above, separately for the two groups.

All independent variables were assessed for multicollinearity with acceptable VIFs <2. Findings with a two-tailed *p* < 0.05 were considered statistically significant; analyses were performed using IBM SPSS Statistics 26 for Windows.

## Results

### Participants

Table 1 presents participant characteristics for all test variables for the total sample (*n* = 385), and stratified by diagnostic group (i.e., NC vs. MCI). On average, participants were approximately 83 years old, were more often female (56%), and had just over 12 years of education. There was a low rate of depressive symptoms in the total sample, and participants self-reported more conscientiousness and openness compared to neuroticism. Group-level differences in participant characteristics for those with NC vs. MCI emerged for several test variables. Compared with the NC participants, those with MCI were older and had higher CVD risk scores, significantly lower global cognition scores, and openness scores. In terms of outcome variables, participants with MCI had significantly higher (worse) self- and informant-reported IADL and significantly lower (worse) performance-based IADL scores.

**TABLE 1:** Characteristics of the sample by total sample (All), normal cognition (NC), and Mild Cognitive Impairment (MCI).

	ALL (*N* = 385)	NC (*N* = 235)	MCI (*N* = 150)	Test statistic	*P*-value
**Predictor variables**					
Age, mean (SD)	83.21 (4.30)	82.81 (4.15)	83.83 (4.47)	*t* = –2.29	**0.023**
Sex—Female, n (%)	217 (56.4)	137 (58.3)	80 (53.3)	χ^2^ = 0.338	0.345
Education (years), mean (SD)	12.05 (3.57)	12.24 (3.58)	11.74 (3.56)	*t* = 1.34	0.180
Arthritis + vision impaired, n (%)	31 (8.1)	15 (6.4)	16 (10.7)	χ^2^ = 0.132	0.178
CVD risk score, mean (SD)	16.46 (3.45)	16.14 (6.78)	16.98 (3.23)	*t* = –2.29	**0.023**
Total no. medications mean (SD)	6.85 (3.36)	6.78 (3.20)	6.97 (3.60)	*t* = –0.54	0.592
^†^Global Cognition, mean (SD)	–0.19 (1.08)	0.33 (0.80)	–1.00 (0.95)	*t* = 14.72	**<0.001**
GDS, mean (SD)	1.43 (0.72)	1.37 (0.71)	1.52 (0.72)	*t* = –1.96	0.051
Neuroticism, mean (SD)	14.52 (6.67)	14.05 (6.53)	15.26 (6.85)	*t* = –1.72	0.087
Openness, mean (SD)	27.55 (5.88)	28.38 (5.62)	26.25 (6.06)	*t* = 3.48	**0.001**
Conscientiousness, mean (SD)	34.14 (5.93)	33.97 (5.87)	34.41 (6.03)	*t* = –0.71	0.478
**Outcome variables**					
Participant Bayer-IADL, mean (SD)	1.52 (0.62)	1.47 (0.51)	1.60 (0.67)	*t* = –2.22	**0.027**
Informant Bayer-IADL, mean (SD)	1.75 (0.83)	1.61 (0.72)	1.95 (0.87)	*t* = –4.13	**<0.001**
STAM score, mean (SD)	30.32 (4.59)	31.78 (3.59)	28.01 (5.15)	*t* = 8.46	**<0.001**

*SD, standard deviation; CVD, cardiovascular disease; GDS, Geriatric Depression Scale; STAM, The Sydney Test of Activities of Daily Living in Memory Disorders.*

*^†^Composite z-score. Bold values significant at p < 0.05.*

### Correlations Between Instrumental Activities of Daily Living Measures

Self-reported IADL scores were positively correlated with informant-reported scores, such that participants who reported more IADL difficulty also had informants who reported more IADL difficulty (*r* = 0.192, *p* < 0.001, *N* = 385). Conversely, performance-based IADL scores were significantly, and negatively, correlated with both self- (*r* = –0.151, *p* = 0.003, *N* = 385) and informant-reported IADL (*r* = –0.294, *p* < 0.001, *N* = 385). However, it is important to note that higher scores on the Bayer-IADL are indicative of worse IADL function whereas higher scores on the STAM indicate better IADL function. In sum, the strongest correlation to emerge was between informant-reported Bayer-IADL and STAM scores. Next, informant-reported, and participant-reported, Bayer-IADL scores emerged as significantly, but weakly, correlated (*r* < 2), as were participant-reported Bayer-IADL and STAM scores, which showed the weakest relationship among the three dependent variables.

### Predictors of Self-Report Bayer-Instrumental Activities of Daily Living

Table 2 presents the results of a three-step hierarchical multiple regression predicting self-report IADL as the dependent variable. At step one, demographic and medical covariates contributed significantly to the regression model, *F*(6, 348) = 2.24, *p* = 0.039, and accounted for 3.7% of the variance in self-report IADL scores. Introducing the cognitive variables at step two contributed 2.8% of additional variance to the model, which was statistically significant, *F*(1, 347) = 10.43, *p* = 0.001. In step three, the addition of the depression and personality variables explained an additional 22.4% of the variance in self-report IADL, which was highly significant, *F*(4, 343) = 27.06, *p* < 0.001. Specifically, higher GDS and neuroticism scores, and lower conscientiousness scores, were related to higher (i.e., worse) self-reported IADL scores, suggesting participants’ appraisals of their own functional ability were influenced by depressive symptoms and certain personality traits, over and above demographics, medical covariates, and global cognition.

**TABLE 2:** Hierarchical linear regression predicting self-report Bayer-IADL.

Variable	β	*t*	sr^2^	*R*	*R* ^2^	Δ*R*^2^
Step 1				0.193	0.037	0.037*
Age	0.072	1.349	0.005			
Sex	–0.075	1.307	0.005			
Education	–0.008	0.158	0.000			
Arthritis + vision impairment	0.140	2.588*	0.019			
CVD risk	0.075	1.323	0.005			
Total no. medications	– 0.055	1.007	0.003			
Step 2				0.256	0.065	0.028**
Age	0.001	0.015	0.000			
Sex	–0.057	1.002	0.003			
Education	0.060	1.050	0.003			
Arthritis + vision impairment	0.136	2.558*	0.018			
CVD risk	0.072	1.292	0.004			
Total no. medications	–0.073	1.353	0.005			
Global cognition	–0.196	3.229**	0.028			
Step 3				0.538	0.290	0.224***
Age	–0.042	0.822	0.001			
Sex	–0.036	0.713	0.001			
Education	0.076	1.430	0.004			
Arthritis + vision impairment	0.062	1.310	0.004			
CVD risk	0.072	1.469	0.004			
Total no. medications	–0.111	2.331*	0.011			
Global cognition	–0.141	2.576*	0.014			
GDS	0.317	6.147***	0.078			
Neuroticism	0.166	3.063**	0.019			
Openness	0.047	0.896	0.002			
Conscientiousness	–0.185	3.639***	0.027			

N = 403; *p < 0.05, **p < 0.01, ***p < 0.001.

*GDS, Geriatric Depression Scale (15-item version); CVD, cardiovascular disease.*

### Predictors of Informant-Report Bayer-Instrumental Activities of Daily Living

Table 3 presents the results of a three-step hierarchical multiple regression predicting informant-report IADL as the dependent variable. Participants’ demographics and medical covariates contributed significantly to the model at step one, *F*(6, 348) = 5.87, *p* < 0.001, and accounted for 9.2% of the variance in informant-reported IADL. The addition of global cognition in step two explained an additional 4.7% of the variance in scores, which was highly significant, *F*(1, 347) = 18.85, *p* < 0.001. In step three, participants’ depression and personality scores accounted for an additional 7.5% of the total model variance, over and above demographic, medical, and cognitive variables. This additional variance was statistically significant, *F*(4, 343) = 8.21, *p* < 0.001, and largely driven by participants’ GDS scores, which uniquely explained 6% of the variation in informant-report IADL scores. These results suggest that while informants’ appraisals of participants’ functional ability are influenced by some variables expected to impact functional ability (e.g., years of education, number of medications, and global cognition scores), they are also significantly impacted by dynamic participant characteristics like depression.

**TABLE 3:** Hierarchical linear regression predicting informant-report Bayer-IADL.

Variable	β	*T*	sr^2^	*R*	*R* ^2^	Δ*R*^2^
Step 1				0.303	0.092	0.092***
Age	0.124	2.400*	0.015			
Sex	–0.127	2.269*	0.013			
Education	–0.131	2.514*	0.016			
Arthritis + vision impairment	0.084	1.598	0.007			
CVD risk	0.045	0.819	0.002			
Total no. medications	0.170	3.238**	0.027			
Step 2				0.372	0.139	0.047***
Age	0.033	0.595	0.001			
Sex	–0.104	1.890	0.009			
Education	–0.043	0.781	0.002			
Arthritis + vision impairment	0.079	1.552	0.006			
CVD risk	0.042	0.774	0.001			
Total no. medications	0.147	2.847**	0.020			
Global cognition	–0.253	4.341***	0.047			
Step 3				0.463	0.214	0.075***
Age	–0.012	0.224	0.000			
Sex	–0.104	1.942	0.009			
Education	–0.065	1.161	0.003			
Arthritis + vision impairment	0.039	0.777	0.001			
CVD risk	0.027	0.520	0.001			
Total no. medications	0.115	2.296*	0.012			
Global cognition	–0.232	4.040***	0.037			
GDS	0.280	5.161***	0.061			
Neuroticism	0.018	0.311	0.000			
Openness	0.095	1.710	0.007			
Conscientiousness	–0.007	0.127	0.000			

N = 355; *p < 0.05, **p < 0.01, ***p < 0.001.

*GDS, Geriatric Depression Scale (15-item version); CVD, cardiovascular disease.*

### Predictors of Performance-Based Instrumental Activities of Daily Living

Table 4 presents the results of a three-step hierarchical multiple regression predicting performance-based (STAM) IADL scores. As before, step one included participant demographics and medical covariates, which contributed significantly to the regression model, *F*(6, 348) = 17.29, *p* < 0.001, and accounted for 23.7% of the variance in performance-based IADL scores. Introducing global cognition at step two explained an additional 23% of the variation in performance-based IADL and this change in R^2^ was highly significant, *F*(1, 347) = 148.36, *p* < 0.001, with global cognition scores alone explaining an additional 12% of the variance, over and above age, sex, and education and medical covariates. Interestingly, the addition of depression and personality in the final step did not contribute significantly to the final regression model, *F*(4, 343) = 0.28, *p* = 0.508. Together, these results suggest that scores on performance-based measures of IADLs are influenced most by participant characteristics like age, sex, education, and cognitive variables, as opposed to current depression or certain personality traits.

**TABLE 4:** Hierarchical linear regression predicting performance-based IADL.

Variable	β	*t*	sr^2^	*R*	*R* ^2^	Δ*R*^2^
Step 1				0.479	0.230	0.230***
Age	–0.304	6.388***	0.090			
Sex	0.165	3.214**	0.023			
Education	0.342	7.136***	0.113			
Arthritis + vision impairment	–0.025	0.520	0.001			
CVD risk	0.017	0.332	0.000			
Total no. medications	–0.092	1.905	0.008			
Step 2				0.679	0.460	0.231***
Age	–0.101	2.333*	0.008			
Sex	0.114	2.622**	0.011			
Education	0.146	3.381**	0.018			
Arthritis + vision impairment	–0.015	0.378	0.000			
CVD risk	0.025	0.582	0.001			
Total no. medications	–0.040	0.986	0.002			
Global cognition	0.562	12.180***	0.231			
Step 3				6.82	0.466	0.005
Age	–0.086	1.932	0.006			
Sex	0.104	2.357*	0.009			
Education	0.145	3.138**	0.015			
Arthritis + vision impairment	–0.009	0.220	0.000			
CVD risk	0.028	0.648	0.001			
Total no. medications	–0.043	1.041	0.002			
Global cognition	0.561	11.839***	0.218			
GDS	–0.026	0.589	0.001			
Neuroticism	0.065	1.386	0.003			
Openness	0.022	0.474	0.000			
Conscientiousness	0.066	1.498	0.003			

N = 355; *p < 0.05, **p < 0.01, ***p < 0.001.

*GDS, Geriatric Depression Scale (15-item version); CVD, cardiovascular disease.*

### *Post-hoc* Analyses of Diagnostic Group Differences

Finally, to explore whether patterns of association differed by diagnostic status (i.e., NC vs. MCI), we re-ran our three hierarchical regressions, using the same steps and covariates as before, separately for the two groups. These results are presented in Supplementary Material. In general, the pattern of results was similar across outcome measures. Supplementary Table 1 presents the results for the self-reported Bayer-IADL. In the fully adjusted model, GDS (NC: β = 0.248, *p* < 0.001; MCI: β = 0.392, *p* < 0.001) and conscientiousness (NC: β = –0.180, *p* = 0.008; MCI: β = –0.174, *p* = 0.040) remained significant for both groups. However, the significant effect of global cognition (β = –0.153, *p* = 0.042) and neuroticism (β = 0.241, *p* = 0.001) appear to be driven mostly by NC participants. Supplementary Table 2 presents the results for the informant-reported Bayer-IADL. In the fully adjusted model, the effect of total number of medications was driven by the NC group (β = 0.185, *p* = 0.008) where the effects of global cognition (β = –0.177, *p* = 0.042) and GDS (β = 0.501, *p* < 0.001) were driven by the MCI group. Openness also emerged as significant for MCI participants (β = 0.222, *p* = 0.010), where this was not significant in the original model. Finally, Supplementary Table 3 presents the results for the performance-based IADL model. In the fully adjusted model, global cognition remained a significant predictor of total score for both groups (NC: β = 0.413, *p* < 0.001; MCI: β = 0.583, *p* < 0.001), though the association with education appears to be driven by the NC group, only (β = 0.215, *p* = 0.001).

## Discussion

This study examined the associations between measures of cognitive function, depressive symptoms, and personality traits and different methods of assessing IADL in a community-dwelling older sample, controlling for demographics and potential medical confounders. When considering the total sample, we found that self-reported worse IADL was associated with worse global cognition scores, depressive symptoms, higher scores on neuroticism, and lower scores on conscientiousness. Worse informant-reported IADL function was also associated with worse global cognition scores and increased depression scores but was not associated with participants’ personality traits. Finally, performance-based IADL scores were associated with age, sex, education, and global cognition, but were not associated with depression or personality traits in any significant way. In addition, the three measures of IADL were only weakly correlated, which may reflect how each measure is variably influenced by demographics, mood, personality traits, and cognition. *Post-hoc* analyses considering diagnostic groups revealed that in some instances associations with the full sample analysis held (e.g., GDS and conscientiousness predicting participant-reported IADL and global cognition predicting performance-based IADL) in other cases differences were driven by one group over the other (e.g., GDS and global cognition predicting informant-reported IADL for MCI only). However, given we were most interested in understanding how these variables across the predementia spectrum influence different measures of functional ability, we focus our attention on the group level analyses.

Our findings that self-reported IADL impairment is significantly associated with higher neuroticism and lower conscientiousness align with other previous reports (Chapman et al., 2007; Suchy et al., 2010; Puente et al., 2015). Given that these personality traits are not significantly associated with informant-reported and performance-based IADL, these findings may imply that individuals with such traits are more prone to over-report IADL impairment. Indeed, individuals who score higher on measures of neuroticism tend to experience more, and ruminate over, negative emotions like stress and anxiety (Widiger and Oltmanns, 2017). As such, individuals with higher neuroticism may tend to over-report IADL impairment due to emotional lability, pessimistic views of oneself, and feelings of vulnerability (Widiger and Oltmanns, 2017). In addition, conscientiousness is characterized by being diligent, organized, self-disciplined, and determined. Previous studies have suggested that individuals with higher conscientiousness may either experience less functional impairment (due to better able to plan and execute tasks),Krueger et al. (2006) or be less willing to admit ADL impairments (Roy et al., 2016; Williams et al., 2017). Our findings indicate that the reverse may also be true, where individuals with low conscientiousness may be less reluctant to reveal IADL impairments and maybe even more prone to over-report impairment. These findings suggest that self-reported IADL may be biased by such personality traits and thus, may not truly reflect the individual’s level of function (Suchy et al., 2010). This has important clinical implications when considering the validity of a patient’s self-reported function.

Our findings further revealed that depression was significantly associated with worse subjective IADL ratings reported by both the participant and their informant, suggesting that individuals with depressive symptoms, and their informants, may tend to overreport IADL impairments. These results support previous research showing depressive symptoms predict worse self-reported IADL performance over time (Ryu et al., 2016; Sutin et al., 2016). However, the direction of this relationship remains unclear. That is, previous research has suggested that depression may precede functional impairment (Kong et al., 2019) as depressive symptoms are highly correlated with loss of energy and motivation, decreased activity, poorer health behaviors, and psychomotor slowing (Lenze et al., 2001; Schillerstrom et al., 2008). On the other hand, declines in functional ability may be the precursor to depression (Schillerstrom et al., 2008) as a loss of independence for daily tasks is highly correlated with lower perceived quality of life and worse self-reported life satisfaction (Meltzer et al., 2012). Only longitudinal studies, however, can determine the impact of depression and its comorbidities on functional decline.

The finding that depressive symptoms were not associated with IADL function as measured by an objective performance-based measure favors the hypothesis that depressive symptoms may be related to overreporting of IADL impairment rather than actual functional decline. One explanation for this relationship is that participants experiencing depressive symptoms may more easily recollect negative instances when they could not perform daily tasks. This is supported by research on the relationship between mood and memory (Williams, 1999). However, it is important to note that participants in our study did not have clinical depression and that indeed many older adults experience depressive symptoms (Dozeman et al., 2010). Thus, to disentangle the complex relationship between increased depression and worse subjectively reported IADL and preserved performance-based objective IADL, a prospective examination of these associations using longitudinal data is required. Nevertheless, our results confirm the importance of screening for depressive symptoms when assessing IADL.

Interestingly, all three measures of IADL were significantly associated with global cognition. However, this association was strongest for objective performance-based IADL ability in comparison to subjectively reported IADL. Nearly 22% of the total variance in performance-based IADL scores was predicted by global cognition compared to less than 2% for self-report and less than 4% for informant-report. Again, this may be due to the impact of depressive symptoms and personality traits on subjective impressions of IADL ability. Taken together, our results suggest that performance-based measures are more reflective of the individual’s cognitive status compared to performance-based measures. This is consistent with previous research which has demonstrated that performance-based IADL measures may be more sensitive in discriminating between cognitive status (Pereira et al., 2010).

Strengths of our study are the relatively large and well-characterized sample, expert consensus clinical diagnosis of NC vs. MCI, the inclusion of important medical covariates, and the three types of IADL measures. To our knowledge, subjective self- and informant-reported IADL and performance-based IADL have not been examined together with depression and personality in one cohort. This study has certain limitations. Firstly, the data analyzed are cross-sectional and provide limited information on whether depressive symptoms are the antecedent or consequences of functional impairment in IADL. Further longitudinal studies should endeavor to explore this issue, particularly relating to performance-based IADL measures. We do not include measures of informant-reported depression, personality, or perceived burden, which may also impact their subjective reports of participants’ IADL ability. A final limitation is that this study only examined individuals with normal cognition or MCI. Thus, the findings do not extend to individuals with dementia. As dementia can have a long prodromal period, future research should look at whether the relationships reported here are similar or different for persons with mild or moderate dementia diagnoses.

In sum, while self-reported IADL function was significantly associated with the participant’s current level of global cognition it was more strongly associated with depressive symptoms and personality traits. Informant-reported IADL was also influenced by depressive symptoms but was not impacted by personality traits and was strongly associated with global cognition. Finally, performance-based IADL scores were not significantly associated with the participant’s depression and personality traits; instead, they were mostly accounted for by the participant’s age, sex, education, and global cognition—all variables known to be associated with functional ability in older adults. We argue that performance-based IADL measures are preferable when trying to accurately assess everyday functional ability and its relationship to cognitive status. Where performance-based measures are not available (e.g., in some clinical settings), informant ratings should be sought as they are less influenced by the participant’s personality and mood compared to self-reports.

## Data Availability Statement

The terms of consent for research participation stipulate that an individual’s data can only be shared outside of the MAS investigators group if the group has reviewed and approved the proposed secondary use of the data. This consent applies regardless of whether data have been de-identified. Access is mediated via a standardised request process managed by the CHeBA Research Bank, who can be contacted at ChebaData@unsw.edu.au, or *via* KN, k.numbers@unsw.edu.au.

## Ethics Statement

The studies involving human participants were reviewed and approved by the University of New South Wales Human Research Ethics Committee. The patients/participants provided their written informed consent to participate in this study.

## Author Contributions

KN designed this study, wrote this manuscript, completed all analyses, and prepared this manuscript for submission. SJ, HB, PS, and BD provided statistical, neuropsychological, and medical guidance, and reviewed this manuscript and revisions. SR provided detailed feedback and guidance at each step of design and manuscript drafting as senior author. All authors contributed to the article and approved the submitted version.

## Conflict of Interest

HB was advisory board member or consultant to Biogen, Nutricia, Roche and Skin2Synapse. He was Medical/Clinical Advisory Board member for Montefiore Homes and Cranbrook Care. The remaining authors declare that the research was conducted in the absence of any commercial or financial relationships that could be construed as a potential conflict of interest.

## Publisher’s Note

All claims expressed in this article are solely those of the authors and do not necessarily represent those of their affiliated organizations, or those of the publisher, the editors and the reviewers. Any product that may be evaluated in this article, or claim that may be made by its manufacturer, is not guaranteed or endorsed by the publisher.
