# Supplementary material for: Instrumental Activities of Daily Living by Subjective and Objective Measures: The Impact of Depression and Personality
Source: Front Aging Neurosci. 2022 Jul 22;14:829544. doi: 10.3389/fnagi.2022.829544 (PMC9353936; doi:10.3389/fnagi.2022.829544)
Supplement: Supplementary file 1 [file Data_Sheet_1.DOCX]

**Release notes for the Sydney Test of Activities of Daily Living in Memory Disorders (STAM)**

The STAM is a performance-based measure of activities of daily living that has been previously validated in the MAS sample (Reppermund et al., 2017).

The scale contains 9 tasks, comprised of 4 items, where each Task is summed on a 4-point scale to a total of 36. Scores can range from 0 to 36, with higher scores indicating better performance (i.e., more intact IADLS).

The scale contains the following 9 Tasks (with corresponding 4 items for each task):

1. **Making a phone call (domain: communication)**

Items: 1. Looks up and finds correct phone number; 2. Correctly writes the number down; 3. Correctly dials the number they have written down; 4. Completes task without prompting*

1. **Putting on a shirt (domain: dressing)**

Items: 1. Puts shirt on correctly; 2. Does up some buttons; 3. Does up all buttons ;4. Undoes buttons to remove

1. **Paying a bill by check (domain: handling finances)**

Items: 1. Correctly fills out bill amount; 2. Correctly fills out payee; 3. Signs cheque; 4. Completes task with no prompting*

1. **Preparing the check for mailing (domain: managing everyday activities)**

Items: 1. Writes correct address; 2. Puts all required components in envelope; 3. Stamps envelope correctly; 4. Completes task with no prompting*

1. **Reading the time and setting an alarm (domain: time orientation)**

Items: 1. States correct time; 2. Sets clock hour correctly; 3. Sets clock minutes correctly; 4. Sets alarm correctly

1. **Managing medications using a dispenser (domain: medication management)**

Items: 1. Medication 1 correct; 2. Medication 2 correct; 3. Medication 3 correct; 4. Medication 4 correct

1. **Choosing items to make a simple recipe (domain: shopping)**

Items: 1. 200g butter; 2. 250g dark chocolate; 3. 6 eggs; 4. Caster sugar

1. **Calculating cost and counting money (domain: counting money)**

Items: 1. Correctly calculates price; 2. Counts out correct money; 3. Correctly calculates change from $20; 4. Correctly calculates price including orange juice and Tim Tams

1. **Recalling activities completed (domain: memory)**

Items: 1. Recalls 1 activity; 2. Recalls 1 activity; 3. Recalls 1 activity; 4. Recalls 1 activity

*total time limit divided by 3 – see notes for adjust STAM time scores

**Related file(s): “M_w4_stam”, “M_w4_stam_adjTime”, “M_w6_stam”, “M_w6_stam_adjTime”**

Two files exist for each wave containing STAM test scores. The first file contains raw STAM task and item scores, as well as the total STAM score adjusted for the number of valid items. The second file contains STAM task scores adjusted for time, as described in the notes for adjusted STAM time scores. This second file does not contain scores for individual items within a task. The adjusted STAM time task scores are unique from the raw STAM task scores only for participants that took longer than the time limit to complete a certain task, or for participants with a missing time (999) on a particular task.

If wanting to examine STAM scores that are adjusted for time, you can use the adjusted STAM time scores. If wanting to adjust task scores for participants who were overtime using a different method, or if wanting to examine raw STAM scores prior to being adjusted for time, regardless of whether they were overtime, you can use the raw and adjusted STAM task and item scores.

**Raw and Adjusted STAM task and item scores**

If a participant refused to perform a given item or was too impaired to complete the activity, this item was scored 0. If a participant had a physical impairment that prevented them from performing the activity, and thus the item was never administered, the item was scored 999.

The STAM_TOTAL variable reflects the unadjusted sum of the individual task scores a participant completed successfully.

The Total_Missing_Tasks variable reflects the number of missing tasks i.e. the number of tasks with a task score of 999, and a Task_NA of 1.

The STAM_OutOf variable reflects the maximum score of valid items. That is, if a participant was only administered 7 of the 9 Tasks due to physical limitations, then the maximum possible score would be 28 instead of 36.

The STAM_AdjTotal reflects the total score adjusted for the maximum possible score and is obtained by dividing STAM_TOTAL by STAM_OutOf.

**Adjusted STAM time scores**

The Task_adj score reflects the task score adjusted for penalties for being overtime, which is calculated using the time limit per item. The time limit per item for a task is calculated by dividing the time limit for the task by the number of timed items within that task. This time limit per item is used to develop time brackets which determine the penalty that the participant’s total task score is subjected to, where being overtime by within one multiple of the time limit per item results in a deduction to the maximum possible score they can attain for the task by 1 point. If a participant is overtime by within two multiples of the time limit per item, there is a deduction to the maximum possible score they can attain by 2 points, and so on.

The number of timed items for tasks 2, 5, 6, 7, 8, and 9 is 4. The number of timed items within tasks 1, 3 and 4 excludes the item of ‘Completes task without prompting’ such that the time limit per item is attained by dividing the time limit by 3 instead of the usual 4, and the maximum possible score a participant can attain for tasks 1, 3 and 4 when considering the time penalty is 3, instead of 4. The score for the ‘Completes task without prompting’ item is then added separately to the adjusted STAM time task score, if the participant gained a score for that item.

This is summarised as follows in Table 1.

**Table 1 Time brackets for overtime participants**

| Task | Time limit (in seconds) | Items | Time limit per item | Total score minus 1 | Total score minus 2 | Total score minus 3 | Total score minus 4 |
| --- | --- | --- | --- | --- | --- | --- | --- |
| 1 | 180 | 1-3 (4 is prompt) | 60 | 181-240 | 241-300 | ≥301 | N/A |
| 2 | 120 | 1-4 | 30 | 121-150 | 151-180 | 181-210 | ≥211 |
| 3 | 210 | 1-3 (4 is prompt) | 70 | 211-280 | 281-350 | ≥351 | N/A |
| 4 | 210 | 1-3 (4 is prompt) | 70 | 211-280 | 281-350 | ≥351 | N/A |
| 5 | 120 | 1-4 | 30 | 121-150 | 151-180 | 181-210 | ≥211 |
| 6 | 210 | 1-4 | 52.5 | 211-262.5 | 263-315.5 | 316-368.5 | ≥369 |
| 7 | 180 | 1-4 | 45 | 181-225 | 226-270 | 271-315 | ≥316 |
| 8 | 270 | 1-4 | 67.5 | 271-337.5 | 338-405 | 406-472.5 | ≥473 |
| 9 | 150 | 1-4 | 37.5 | 151-187.5 | 188-225 | 226-262.5 | ≥263 |

If a participant has a valid task score, but the time for the task is coded as 999, the task score is unchanged for the raw STAM scores, but for the adjusted STAM time scores, the task score is re-coded into 999.

The TOTAL_STAM_adjTime variable reflects the sum of the adjusted STAM time task scores as described above.

Reference: Reppermund, S.*, et al.* (2017). Performance-Based Assessment of Instrumental Activities of Daily Living: Validation of the Sydney Test of Activities of Daily Living in Memory Disorders (STAM). *Journal of the American Medical Directors Association* **18**, 117-122
