# Supplementary material for: Instrumental Activities of Daily Living by Subjective and Objective Measures: The Impact of Depression and Personality
Source: Front Aging Neurosci. 2022 Jul 22;14:829544. doi: 10.3389/fnagi.2022.829544 (PMC9353936; doi:10.3389/fnagi.2022.829544)
Supplement: Supplementary file 2 [file Data_Sheet_2.DOCX]

**Sydney Test of Activities of Daily Living in Memory Disorders (STAM)**

This next task is called the Sydney Test of Activities of Daily Living in Memory Disorders (STAM). The STAM is a little different from the other activities we’ve done today; it’s quite practical and looks at everyday living skills like making phone calls and

paying bills. I’d like you to try and complete these activities as close as possible to the way you would in real life.

### Task 1 – Making a phone call (3 minutes)

- Phone book, Telephone (not connected), Pen and paper with name/address

I would like you to make a phone call for me. First, I would like you to find a number in the phone book and write it down. Then I would like you to dial the number on this phone. The phone is not connected but I would like you to make the phone call as you would normally. The phone number I would like you to look up is:

Southern Cross Dry Cleaners Pty Ltd

**3 Albion St Waverley (**The correct number is (02) 9389 9357)

**Please write the number down on this sheet of paper.** Hand the participant the sheet of paper with the name and address on it and a space for the number. Begin Timing.

**Time to complete task 1**

| **Item** | **Score** | |
| --- | --- | --- |
| 1. Looks up and finds correct phone number **(9389 9357)** | 1 | 0 |
| 2. Correctly writes the number down | 1 | 0 |
| 3. Correctly dials the number they have written down | 1 | 0 |
| 4. Completes task without prompting | 1 | 0 |
| Total score (max = 4) |  | |

General prompts used:

 I’d like you to do what you think is right

 I’d like you to do it as you would in real life

Other prompts used:

### Task 2 – Putting on a shirt (2 minutes)

- Large shirt with large buttons – one female and one male shirt (button differently). Shirts will need to be laundered periodically.

**Next, I would like you to put on this shirt over your clothes and do up all of the buttons.** Hand the participant the folded shirt and observe as they put the shirt on. Begin timing.

Once the shirt is on and buttoned up, say: **Now please remove the shirt.** Observe whether they remove the shirt by undoing the buttons or just pulling over head.

**Time to complete task 2**

| **Item** | **Score** | |
| --- | --- | --- |
| 1. Puts shirt on correctly (right side out and arms through correct holes) | 1 | 0 |
| 2. Does up some buttons (give automatically if all done up) | 1 | 0 |
| 3. Does up all buttons | 1 | 0 |
| 4. Undoes buttons to remove | 1 | 0 |
| Total score (max = 4) |  | |

General prompts used:

 I’d like you to do what you think is right

 I’d like you to do it as you would in real life

Other prompts used:

**Task 3 – Pay a Bill by cheque (3.5 minutes)**

- Bill, cheque, pen, envelope,1 stamp – all placed on table in front of participant

For this activity, I would like you to pay this gas bill by cheque and get it ready to post. I would like you to complete the cheque and prepare the envelope for posting as you would normally. Please let me know when you are finished. Begin timing with the stopwatch.

**Time to complete task 3**

| **Item** | **Score** | |
| --- | --- | --- |
| 1. Correctly fills out bill amount **($92.10)** | 1 | 0 |
| 2. Correctly fills out payee **(Energy Australia)** | 1 | 0 |
| 3. Signs cheque | 1 | 0 |
| 4. Completes task with no prompting | 1 | 0 |
| Total score (max = 4) |  | |

General prompts used:

 I’d like you to do what you think is right

 I’d like you to do it as you would in real life

Other prompts used:

### Task 4 – Prepare the bill for mailing (3.5 minutes)

Participant then prepares the envelope to post the bill including addressing, stamp, inserting cheque and closing envelope. Begin timing with the stopwatch.

**Time to complete task 4**

| **Item** | **Score** | |
| --- | --- | --- |
| 1. Writes correct address **(Locked Bag 33, Silverwater, NSW, 2128)** | 1 | 0 |
| 2. Puts all required components in envelope **(cheque)** | 1 | 0 |
| 3. Stamps envelope correctly | 1 | 0 |
| 4. Completes task with no prompting | 1 | 0 |
| Total score (max = 4) |  | |

General prompts used:

 I’d like you to do what you think is right

 I’d like you to do it as you would in real life

 Is it completely ready to post?

Other prompts used:

### Task 5 – Time Orientation (2 minutes)

- Clock with alarm (Clock should be pre-set to 4.30 and alarm hand pointing to 3)

Begin timing with the stopwatch*.* **Now I would like you to tell me the time on this clock.** Show them the clock (*correct time is 4.30)***.**

Next, I would like you to set the time. The time I would like you to set the clock for is 7.25. Please let me know when you are finished. Once they have set the time, say:

Now I would like you to set the alarm. The time that you should set the alarm to is 6.00.

**Time to complete task 5**

| **Item** | **Score** | |
| --- | --- | --- |
| 1. States correct time **(4.30)** | 1 | 0 |
| 2. Sets clock hour correctly **(7)** | 1 | 0 |
| 3. Sets clock minutes correctly **(7.25)** | 1 | 0 |
| 4. Sets alarm correctly **(pointing to 6)** | 1 | 0 |
| Total score (max = 4) |  | |

General prompts used:

 I’d like you to do what you think is right

 I’d like you to do it as you would in real life

Other prompts used:

### Task 6 – Medication Management (3.5 minutes)

- 4 medication bottles filled with tablets with instructions for use, daily medication dispenser with 4 sections for the day (morning, noon, evening and bed)

Here we have four bottles of medications. The label on each bottle tells you when each medication should be taken and how much. I would like you to fill this medication dispenser with all of the medication that you would need to take for one day according to the instructions on each bottle. Please let me know when you are finished. Begin timing with the stopwatch.

**Time to complete task 6**

| **Item** | **Score** | |
| --- | --- | --- |
| 1. Medication 1 correct **(Blue: 1 noon, 1 bedtime)** | 1 | 0 |
| 2. Medication 2 correct **(Amber: 2 morning, 2 noon, 2 evening)** | 1 | 0 |
| 3. Medication 3 correct **(Red: 2 morning, 2 evening)** | 1 | 0 |
| 4. Medication 4 correct **(Black: 1 bedtime)** | 1 | 0 |
| Total score (max = 4) |  | |

General prompts used:

 I’d like you to do what you think is right

 I’d like you to do it as you would in real life

Other prompts used:

### Task 7 – Choose items to make a simple recipe (3 minutes)

- Simple recipe, grocery poster, money, purse, paper and pen (for calculations, if needed).

Here is a simple recipe. I would like you to choose the ingredients that you would need to make this recipe from this poster. Can you please tell me which of these ingredients you need to buy? Begin timing with the stopwatch*.*

**Time to complete task 7**

| **Item** | **Score** | |
| --- | --- | --- |
| 1. 200g butter | 1 | 0 |
| 2. 250g dark chocolate | 1 | 0 |
| 3. 6 eggs | 1 | 0 |
| 4. Caster sugar | 1 | 0 |
| Total score (max = 4) |  | |

General prompts used:

 I’d like you to do what you think is right

 I’d like you to do it as you would in real life

 I’d like you to do what you would normally in a real shop

Other prompts used:

### Task 8 – Calculating cost and counting money (4.5 minutes)

1. Begin timing with the stopwatch. **Using the prices on this poster, can you calculate the total cost of the four items (butter, dark chocolate, 6 eggs and caster sugar) that you need to buy? You can choose whether to calculate it in your head or by writing it down.**
2. If they correctly calculate the cost, say: **Can you now please count out the correct money from the purse to pay for the items** *(correct amount is $12.50).*

If they do not correctly calculate the cost, say: **Can you now please count out $12.50 from this purse?**

*Use $12.50 as the starting point for all further calculations.*

1. Once they have counted out the money ask**: If you were paying with a $20 note, can you tell me how much change you would receive?** *(Correct answer is $7.50).*
2. **Starting at $12.50, what would the total cost be if you also bought some orange juice and some Tim Tams?**

*(Answer is $19.50)*

**Time to complete task 8**

| **Item** | | **Score** | |
| --- | --- | --- | --- |
| 1. Correctly calculates price **($12.50)** | | 1 | 0 |
| 2. Counts out correct money | | 1 | 0 |
| 3. Correctly calculates change from $20 **($7.50)**  Participants answer: |  | 1 | 0 |
| 4. Correctly calculates price including orange juice  and Tim Tams **($19.50)** Participants answer: |  | 1 | 0 |
| Total score (max = 4) | |  | |

General prompts used:

 I’d like you to do what you think is right

 I’d like you to do it as you would in real life

 I’d like you to do what you would normally in a real shop

Other prompts used:

### Task 9 – Practical Recall (2.5 minutes)

- Ensure all items are packed away before the recall task to avoid giving the participant any visual prompts.

For the last part of the task, I would like you to tell me about four of the activities that we have completed for the STAM. You don’t have to give them in the order in which we did them, just any four activities that we have done. Begin timing with the stopwatch.

**Time to complete task 8**

| **Item** | **Score** | |
| --- | --- | --- |
| 1. Recalls 1 activity | 1 | 0 |
| 2. Recalls 1 activity | 1 | 0 |
| 3. Recalls 1 activity | 1 | 0 |
| 4. Recalls 1 activity | 1 | 0 |
| Total score (max = 4) |  | |

**STAM Total Scores**

| **Domain** | **Item** | **Score** |
| --- | --- | --- |
| 1. Communication | Phone call | **/4** |
| 2. Dressing | Putting on shirt | **/4** |
| 3. Handling Finances | Paying a bill | **/4** |
| 4. Managing everyday activities | Mailing bill | **/4** |
| 5. Orientation | Time orientation | **/4** |
| 6. Medication Management | Medication dispensing | **/4** |
| 7. Shopping Skills | Choosing items | **/4** |
| 8. Counting Money | Calculating/counting money | **/4** |
| 9. Memory – Recall | Practical recall | **/4** |
| Total Score | | **/36** |

# Southern Cross Dry Cleaners Pty Ltd 3 Albion St Waverley

Phone number:

#
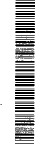
Gas Account


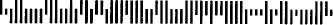


**S. PARTICIPANT**

**22-32 KING ST, RANDWICK, NSW 2031**

Account Number 3333 3333 333

Enquiries 133 333

Gas Supply Faults 133 333


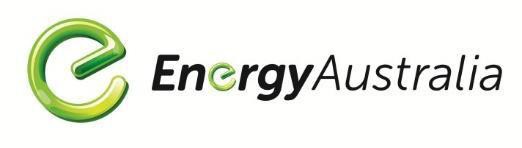


Emergencies ( 24 hours )

133 333


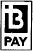

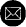

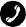

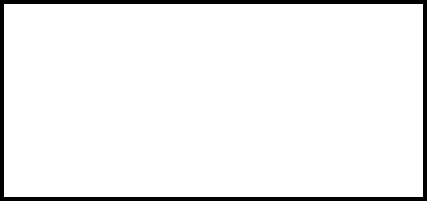

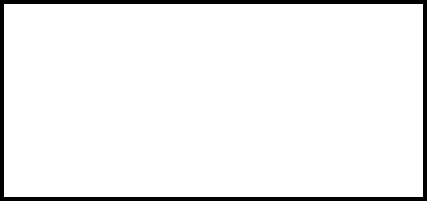

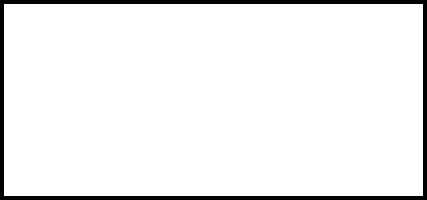


|  |  | | |
| --- | --- | --- | --- |
| Service Address 22-32 King St Randwick, NS | |  |  |
| Tax Invoice Issue date 15 Jan 2019 | |  | **Due Date** |
|  | |  | **30 June 2019** |
| **Gas account summary 1 July 2018 to 31 December 20** | |  |  |
|  | |  | **Total amount payable** |
|  | | $118.85 | **$92.10** |
|  | | $118.85 |  |
|  | | **$0.00** |  |
| Current charges ( inc. GST ) | $92.10 | | |
| **Total amount due ( inc. GST )** | **$92.10** | | |
|  |  | | |
| **Gas Payment Options** | 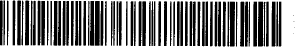 | | |
| **BPAY**  Biller Code: 000000  Ref: 3333 3333 3333  BPAY this payment via internet or phone banking | **Due date** | | **30 June 2019** |
| **Phone**  Call 1300 333 333 to pay by Mastercard or Visa. | **Total Amount due $92.10** | | |
| Ref: 3333 3333 3333 |  | | |
| **Cheque / Mail**  Please post your cheque payable to:  Energy Australia, Locked Bag 33, SILVERWATER NSW, 2128 |  | | |

# Flourless chocolate cake


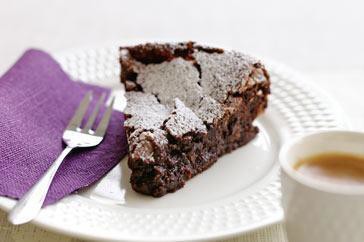


## Ingredients (serves 8)

- 200g butter, chopped
- 250g dark chocolate, chopped
- 6 eggs, separated
- 1 cup caster sugar

## Method

- 1. Preheat oven to 160°C/140°C fan-forced. Grease a 6cm-deep, 22cm (base) round cake pan. Line base and side with baking paper.
  2. Place butter and chocolate in a heatproof bowl over a pan of simmering water (make sure bowl doesn't touch water). Stir over low heat until smooth.
  3. Using an electric mixer, beat egg yolks and 1/2 cup sugar for 5 minutes or until thick and creamy. Beat egg whites in a separate bowl until soft peaks form. Gradually beat in remaining sugar until dissolved.
  4. Add chocolate mixture to egg yolk mixture. Mix to combine. Add one- quarter of the egg white mixture. Mix well. Fold in remaining egg white mixture. Pour into prepared pan. Bake for 50 minutes or until top is firm to touch and a skewer inserted into centre has moist crumbs clinging. Cool cake in pan. Serve.

Dust with icing sugar mixture.
