# Supplementary material for: Instrumental Activities of Daily Living by Subjective and Objective Measures: The Impact of Depression and Personality
Source: Front Aging Neurosci. 2022 Jul 22;14:829544. doi: 10.3389/fnagi.2022.829544 (PMC9353936; doi:10.3389/fnagi.2022.829544)
Supplement: Supplementary file 3 [file Data_Sheet_3.docx]

**Explanatory notes for MAS cognitive domain and global cognition scores.**

**Cognitive Domain Measures**

The cognitive domain measures are attention/processing speed, memory, verbal memory, language, visuo-spatial ability and executive function. Most standardised neuropsychological tests measure multiple cognitive processes, so tests can be classified under more than one domain. In the literature there is no absolute consistency in allocating tests/measures to specific domains. For example, letter fluency (FAS) is sometimes classified as a test of language and sometimes as a test of executive function. The cognitive domain scores were calculated as the average of the quasi Z-scores of the component tests. Each score was again transformed so that the mean and standard deviations for the baseline reference groups were 0 and 1.

**Global Cognition Measures**

Separate global cognition scores for each wave were calculated as the average of the cognitive domain scores for the corresponding wave. These scores were again transformed so that the mean and standard deviations for the baseline reference groups were 0 and 1. Details of the calculation of domain and global cognition scores are given below. Please note that none of the data presented in this file have been adjusted for age, sex or education. So, it is advised that these be included as control variables in analyses investigating relationships with these variables.

**Calculation of Cognitive Domain Scores**

1. Means and SDs of baseline tests for participants who were in the particular reference group (“normal” or “healthy”) were calculated

2. "Quasi Z scores" were calculated for all individual tests using means and SDs of those in the particular reference group at baseline (ie for all measures, means of the baseline group were subtracted, and the result divided by the SDs of the reference group at baseline). Signs of TMTA and TMTB were reversed so that more positive scores represent better performances, as is the case for all other tests.

3. Composite domain scores were first calculated for each participant as the mean of available quasi Z-scores of tests comprising each domain.

4. For the domains comprising just 2 tests (Attention/Processing Speed, Language and Executive Functions), mean scores were retained only if scores for both tests were present.

5. For memory and verbal memory composites, if more than one component test was missing, then composite scores were not retained.

6. In addition, for verbal memory composite, if the logical memory test was missing then the composite was not retained. (So, a composite was calculated if one of the other tests was missing, but not the logical memory test.)

7. Final domain scores were obtained as quasi Z-scores of the above composites, so that means and SDs of domain scores for the particular reference group at baseline were equal to 0 and 1.

**Calculation of Global Cognition Scores**

1. Global cognition composite scores were first calculated as the average of all composite domain scores except verbal memory (as obtained in step 3 above). For the purpose of calculating global cognition scores, domain scores were calculated for all cases, no matter how many tests were present.

2. Global cognition scores were retained if:

a) there were no missing domain composite scores (i.e. there was at least one test per domain present) OR

b) there was only one domain composite score missing AND there were at least 8 of the 12 tests present.

3. Final global cognition scores were standardized by transforming to quasi Z-scores, so that the mean and SD of the global cognition scores for ESB normals at baseline was 0 and 1.

Note: The term “quasi Z-score” was used above to emphasise that the calculation was done using the mean and standard deviation of the reference group at baseline, not of the whole sample at the wave that measure was taken. However, this is not a commonly used term, and it is suggested that for publications you can state that test or domain scores “… were standardized as Z-scores calculated using the mean and standard deviations of a normative reference group at baseline.”
