# Supplementary material for: Instrumental Activities of Daily Living by Subjective and Objective Measures: The Impact of Depression and Personality
Source: Front Aging Neurosci. 2022 Jul 22;14:829544. doi: 10.3389/fnagi.2022.829544 (PMC9353936; doi:10.3389/fnagi.2022.829544)
Supplement: Supplementary file 4 [file Data_Sheet_4.docx]

**Neuropsychological tests used to calculate cognitive domains and global cognition scores.**

| **Cognitive Domain** | **Test** |
| --- | --- |
| Attention/Processing speed | Digit Symbol-Coding (Wechsler, 1997a)  Trail Making Test (TMT) A (Strauss, Sherman, & Spreen, 2006) |
| Memory | Logical Memory Story A delayed recall (Wechsler, 1997b)  Rey Auditory Verbal Learning Test (RAVLT)(Strauss, et al., 2006)  RAVLT total learning; sum of trials 1-5  RAVLT short-term delayed recall; trial 6  RAVLT long-term delayed recall; trial 7  Benton Visual Retention Test recognition (Benton, Sivan, & Spreen, 1996) |
| Verbal Memory | As above, but not including the Benton Visual Retention Test. |
| Language | Boston Naming Test – 30 items (Kaplan, 2001)  Semantic Fluency (Animals) (Strauss, et al., 2006) |
| Visuo-spatial | Block Design (Wechsler, 1981) |
| Executive function | Controlled Oral Word Association Test (FAS) (Strauss, et al., 2006)  Trail Making Test (TMT) B (Strauss, et al., 2006) |

**Test References**

Benton, A. L., Sivan, A. B., & Spreen, O. (1996). *Der Benton Test* (7th ed.). Bern: Huber.

Kaplan, E. (2001). *The Boston Naming Test*. Philadelphia: Lippincott Williams Wilkins.

Nelson, H. E., & Willison, J. (1991). *National Adult Reading Test (NART): Test manual* (2nd ed.): Windsor,UK: NFER Nelson.

Strauss, E., Sherman, E. M. S., & Spreen, O. (2006). *A Compendium of Neuropsychological Tests: Administration, Norms, and Commentary* (3rd ed.). New York: Oxford University Press.

Wechsler, D. (1981). *WAIS-R manual*. New York: The Psychological Corporation.

Wechsler, D. (1997a). *Wechsler Adult Intelligence Scale-III*. San Antonio: The Psychological Corporation.

Wechsler, D. (1997b). *Wechsler Memory Scale. Third edition manual.* San Antonio: The Psychological Corporation.
