# Supplementary material for: Instrumental Activities of Daily Living by Subjective and Objective Measures: The Impact of Depression and Personality
Source: Front Aging Neurosci. 2022 Jul 22;14:829544. doi: 10.3389/fnagi.2022.829544 (PMC9353936; doi:10.3389/fnagi.2022.829544)
Supplement: Supplementary file 5 [file Table_1.DOCX]

| Variable | β | | *t* | | *p* | |
| --- | --- | --- | --- | --- | --- | --- |
| Step 1 | NC | MCI | NC | MCI | NC | MCI |
| Age | .013 | .128 | .191 | 1.492 | .849 | .138 |
| Sex | -.027 | -.110 | -.366 | -1.188 | .714 | .237 |
| Education | .030 | -.037 | .424 | -.432 | .672 | .667 |
| Arthritis + vision  impairment | .101 | .191 | 1.440 | 2.178 | .151 | **.031*** |
| CVD risk | .048 | .115 | .650 | 1.243 | .516 | .216 |
| Total no. medications | .010 | -.127 | .148 | -1.455 | .883 | .148 |
| Step 2 |  |  |  |  |  |  |
| Age | -.070 | .079 | -.927 | -.490 | .355 | .391 |
| Sex | -.001 | -.109 | -.020 | .861 | .984 | .242 |
| Education | .111 | .018 | 1.454 | -1.175 | .148 | .844 |
| Arthritis + vision  impairment | .104 | .187 | 1.504 | .197 | .134 | **.034*** |
| CVD risk | .072 | .107 | .983 | 2.143 | .327 | .247 |
| Total no. medications | -.020 | -.142 | -.283 | 1.163 | .777 | .108 |
| Global cognition | -.211 | -.143 | -2.588 | -1.620 | **.010**** | .149 |
| Step 3 |  |  |  |  |  |  |
| Age | -.055 | -.042 | -.819 | -.487 | .414 | .627 |
| Sex | -.002 | -.069 | -.035 | -.828 | .972 | .409 |
| Education | .103 | .060 | 1.471 | .635 | .143 | .527 |
| Arthritis + vision  impairment | .045 | .082 | .736 | 1.030 | .463 | .305 |
| CVD risk | .084 | .065 | 1.308 | .786 | .192 | .433 |
| Total no. medications | -.074 | -.148 | -1.174 | -1.903 | .242 | .059 |
| Global cognition | **-.153** | -.134 | -2.049 | -1.528 | **.042** | .129 |
| GDS | **.248** | **.392** | 3.661 | 4.605 | **.000***** | **.000***** |
| Neuroticism | **.241** | .085 | 3.323 | .974 | **.001**** | .332 |
| Openness | .052 | .053 | .771 | .605 | .442 | .546 |
| Conscientiousness | **-.180** | **-.174** | -2.674 | -2.076 | **.008**** | **.040*** |

**Supplementary Table 1 (S1). Hierarchical linear regression predicting self-report Bayer-IADL as a function of diagnosis.**

*Note*. N = 403; *p < .05, **p < .01, ***p< .001

GDS = Geriatric Depression Scale (15-item version); CVD = cardiovascular disease

| Variable | β | | *t* | | *p* | |
| --- | --- | --- | --- | --- | --- | --- |
| Step 1 | NC | MCI | NC | MCI | NC | MCI |
| Age | .072 | .155 | 1.080 | 1.835 | .282 | .069 |
| Sex | -.092 | -.128 | -1.277 | -1.392 | .203 | .166 |
| Education | **-.064** | -.186 | -.944 | -2.215 | .346 | **.029*** |
| Arthritis + vision  impairment | .116 | .046 | 1.716 | .531 | .088 | .596 |
| CVD risk | -.031 | .119 | -.434 | 1.300 | .664 | .196 |
| Total no. medications | **.231** | .113 | 3.413 | 1.307 | **.001**** | .194 |
| Step 2 |  |  |  |  |  |  |
| Age | .012 | .105 | .165 | 1.160 | .869 | .248 |
| Sex | -.073 | -.126 | -1.015 | -1.379 | .311 | .170 |
| Education | -.006 | -.129 | -.078 | -1.413 | .938 | .160 |
| Arthritis + vision  impairment | .118 | .042 | 1.760 | .488 | .080 | .626 |
| CVD risk | -.013 | .111 | -.189 | 1.217 | .850 | .226 |
| Total no. medications | .209 | .098 | 3.067 | 1.129 | **.002**** | .261 |
| Global cognition | -.152 | -.147 | -1.921 | -1.514 | .056 | .132 |
| Step 3 |  |  |  |  |  |  |
| Age | .008 | -.012 | .104 | -.138 | .917 | .890 |
| Sex | -.076 | -.114 | -1.048 | -1.401 | .296 | .164 |
| Education | -.013 | -.196 | -.174 | -2.128 | .862 | **.035** |
| Arthritis + vision  impairment | .096 | -.047 | 1.421 | -.604 | .157 | .547 |
| CVD risk | -.011 | .025 | -.162 | .306 | .872 | .760 |
| Total no. medications | **.185** | .073 | 2.698 | .952 | **.008**** | .343 |
| Global cognition | -.124 | **-.177** | -1.520 | -2.050 | .130 | **.042*** |
| GDS | .133 | **.501** | 1.797 | 5.986 | .074 | **.000***** |
| Neuroticism | .061 | -.093 | .778 | -1.078 | .437 | .283 |
| Openness | .021 | **.222** | .290 | 2.599 | .772 | **.010**** |
| Conscientiousness | -.035 | .030 | -.481 | .367 | .631 | .714 |

**Supplementary Table 2 (S2). Hierarchical linear regression predicting informant-report Bayer-IADL as a function of diagnosis.**

*Note*. N = 355; *p < .05, **p < .01, ***p< .001

GDS = Geriatric Depression Scale (15-item version); CVD = cardiovascular disease

| Variable | β | | *t* | | *p* | |
| --- | --- | --- | --- | --- | --- | --- |
| Step 1 | NC | MCI | NC | MCI | NC | MCI |
| Age | **-.295** | **-.271** | -4.814 | -3.433 | **.000***** | **.001**** |
| Sex | .**165** | .158 | 2.473 | 1.847 | **.014*** | .067 |
| Education | **.356** | **.319** | 5.675 | 4.082 | **.000***** | **.000***** |
| Arthritis + vision  impairment | -.042 | -.007 | -.670 | -.085 | .504 | .932 |
| CVD risk | .120 | -.066 | 1.845 | -.779 | .066 | .437 |
| Total no. medications | -.067 | -.157 | -1.070 | -1.959 | .286 | .052 |
| Step 2 |  |  |  |  |  |  |
| Age | **-.139** | -.072 | -2.202 | -1.023 | **.029*** | .308 |
| Sex | .116 | **.151** | 1.850 | 2.124 | .066 | **.036*** |
| Education | **.203** | .095 | 3.173 | 1.341 | **.002**** | .182 |
| Arthritis + vision  impairment | -.048 | .009 | -.820 | .127 | .413 | .899 |
| CVD risk | .075 | -.035 | 1.227 | -.492 | .221 | .624 |
| Total no. medications | -.010 | -.097 | -.163 | -1.449 | .870 | .150 |
| Global cognition | **.400** | **.582** | 5.829 | 7.681 | **.000***** | **.000***** |
| Step 3 |  |  |  |  |  |  |
| Age | -.144 | -.021 | -2.251 | -.291 | .025 | .772 |
| Sex | .112 | .118 | 1.764 | 1.658 | .079 | .100 |
| Education | .**215** | .035 | 3.242 | .435 | **.001**** | .665 |
| Arthritis + vision  impairment | -.047 | .047 | -.809 | .690 | .420 | .491 |
| CVD risk | .079 | -.018 | 1.278 | -.254 | .203 | .800 |
| Total no. medications | -.012 | -.109 | -.196 | -1.625 | .845 | .107 |
| Global cognition | **.413** | **.583** | 5.802 | 7.775 | **.000***** | **.000***** |
| GDS | .033 | -.134 | .510 | -1.843 | .611 | .068 |
| Neuroticism | .037 | .108 | .531 | 1.442 | .596 | .152 |
| Openness | -.045 | .145 | -.702 | 1.945 | .483 | .054 |
| Conscientiousness | .113 | .024 | 1.760 | .332 | .080 | .740 |

**Supplementary Table 3 (S3). Hierarchical linear regression predicting performance-based IADL as a function of diagnosis.**

*Note*. N = 355; *p < .05, **p < .01, ***p< .001

GDS = Geriatric Depression Scale (15-item version); CVD = cardiovascular disease
